# Meta-Analysis of Cytochrome P-450 2C9 Polymorphism and Colorectal Cancer Risk

**DOI:** 10.1371/journal.pone.0049134

**Published:** 2012-11-07

**Authors:** Shuo Liang, Jingsong Hu, Weijun Cao, Sanjun Cai

**Affiliations:** 1 Department of Respiratory, Pulmonary Hospital of Shanghai, Tongji University School of Medicine, Shanghai, People’s Republic of China; 2 Department of Colorectal Cancer, Shanghai Cancer Center, Fudan University School of Medicine, Shanghai, People’s Republic of China; Gentofte University Hospital, Denmark

## Abstract

**Background:**

CYP2C9 encodes a member of the cytochrome P450 superfamily of enzymes which play a central role in activating and detoxifying many carcinogens and endogenous compounds thought to be involved in the development of colorectal cancer (CRC). In the past decade, the relationship between CYP2C9 common polymorphisms (R144C and I359L) and CRC has been reported in various ethnic groups; however, these studies have yielded contradictory results. To investigate this inconsistency, we performed this meta-analysis.

**Methods:**

Databases including Pubmed, EMBASE, Web of Science and China National Knowledge Infrastructure (CNKI) were searched to find relevant studies. Odds ratios (ORs) with 95% confidence intervals (CIs) were used to assess the strength of association.

**Results:**

A total of 13 articles involving 9,463 cases and 11,416 controls were included. Overall, the summary odds ratio of CRC was 0.98 (95% CI: 0.89−1.06) and 0.99 (95% CI: 0.87−1.14) for CYP2C9 144C and 359L alleles, respectively. No significant results were observed using dominant or recessive genetic model for these polymorphisms. In the stratified analyses according to ethnicity and sex, no evidence of any gene-disease association was obtained.

**Conclusions:**

This meta-analysis suggests that the CYP2C9 may not be associated with colorectal cancer development.

## Introduction

Colorectal cancer (CRC), second only to lung cancer, is a major cause of cancer death in the western world [1]. Despite much investigation, the causes are not yet fully understood. The marked regional differences of CRC incidence rates implicate the combined influence of genetic predisposition and local environmental factors such as local carcinogen exposure and diet [2]–[4].

Diverse xenobiotic-metabolizing enzymes that are capable of activating carcinogens and mutagens are expressed in human intestinal epithelium [5], [6]. Among them, cytochrome P450 (CYP) enzymes play a key role in the metabolism of xenobiotics. The CYP2C enzyme subfamily accounts for about 20% of the total CYP enzymes in human liver, CYP2C9 being the most abundant [7], [8]. CYP2C9 is involved in both the activation of dietary carcinogens and mutagens, liver metabolism and local metabolism in intestinal epithelium may occur. Since CRC risk is epidemiologically linked to dietary habits, CYP2C9 gene may be a good candidate for genetics studies on CRC. Several important single nucleotide polymorphisms have been identified in the CYP2C9 gene. Two coding-region CYP2C9 variants (Arg144Cys and Ile359Leu) encode three common polymorphisms: the wild-type CYP2C9*1 allele, and two variant *2, and *3 alleles identified in Caucasians [9]–[12]. In vitro analyses have shown substantial variation in CYP2C9 metabolic capacity with the variant *2 and *3 alleles associated with 30% and 80% lower enzymatic activity, respectively, when compared with the wild-type *1 allele [13], [14].

Despite the biological plausibility of CYP2C9 functional polymorphisms as a modulator of CRC susceptibility, previously inconsistent results have appeared in the literature. Published studies have generally been restricted in terms of sample size and ethnic diversity, and individual studies may have insufficient power to achieve a comprehensive and reliable conclusion. We therefore performed a meta-analysis of the published studies to clarify this inconsistency and to establish a comprehensive picture of the relationship between CYP2C9 and CRC.

## Materials and Methods

### Literature Search Strategy

Genetic association studies published before the end of Apr. 2012 on CRC and polymorphisms within CYP2C9 gene were identified through a search of PubMed, Web of Science, EMBASE and CNKI (Chinese National Knowledge Infrastructure) without language restrictions. Search term combinations were keywords relating to the CYP2C9 gene (e.g., “cytochrome p450 2C9”, “cytochrome p450 IIC9”, and “CYP2C9”) in combination with words related to CRC (e.g., “colorectal”, “colon”, “rectal” combined with “cancer” or “carcinoma” or “tumor” or “neoplasms”) and polymorphism or variation. The search was supplemented by reviews of reference lists for all relevant studies and review articles. The major inclusion criteria were (a) original papers containing independent data, (b) case–control or cohort studies and (c) genotype distribution information or odds ratio (OR) with its 95% confidence interval (CI) and P-value. The major reasons for exclusion of studies were (a) overlapping data and (b) case-only studies, family-based studies and review articles.

### Data Extraction

For each study, the following data were extracted independently by two authors: first author’s surname, year of publication, diagnosis criterion, age, sex, ethnicity, Hardy–Weinberg equilibrium (HWE) status, genotyping method, source of control, total number of cases and controls and genotype frequency in cases and controls. The results were compared, and disagreements were discussed among all authors and resolved with consensus.

### Statistical Methods

Odds ratio (OR) with 95% confidence intervals (CIs) was used to assess the strength of association between the CYP2C9 gene polymorphism and CRC risk. The per-allele OR of the risk allele of these polymorphisms mentioned above was compared between cases and controls. Additional pooled estimates were also given with corresponding results under dominant and recessive genetic models. Cochran’s chi-square-based Q statistic test was performed in order to assess possible heterogeneity between the individual studies and thus to ensure that each group of studies was suitable for meta-analysis. ORs were pooled according to the method of DerSimonian and Laird that takes into account the variation between studies, and 95% CI were constructed using Woolf’s method [15], [16]. The Z test was used to determine the significance of the pooled OR. Sensitivity analyses were performed to assess the stability of the results, namely, a single study in the meta-analysis was deleted each time to reflect the influence of the individual data set to the overall OR. Publication bias was assessed using Egger’s test [17] and Begg’s funnel plots [18]. All P values are two-sided, and P<0.05 were considered statistically significant. Statistical analyses were done with Stata (version 10.0).

## Results

### Characteristics of Studies

**Figure 1:**
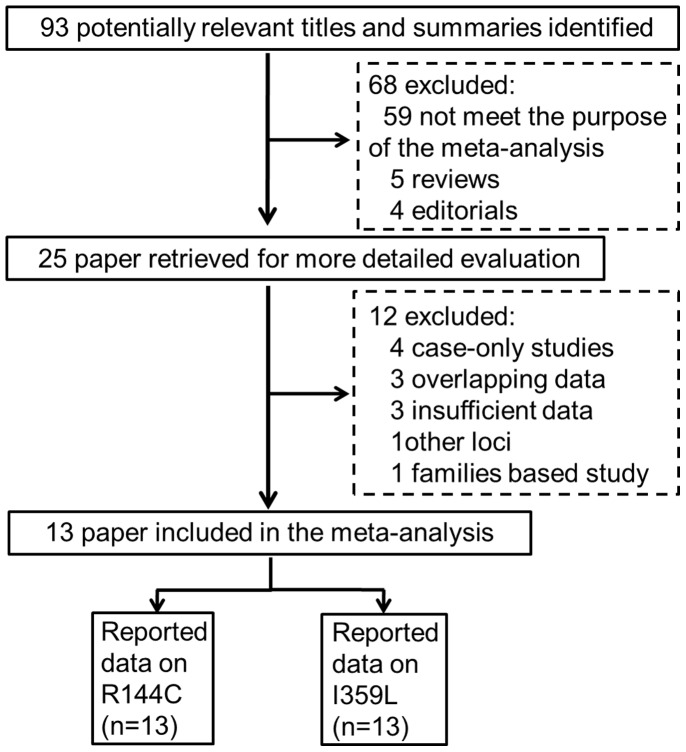
The flow chart of the included studies.

The combined search yielded 93 references. Study selection process was shown in Figure 1. Finally, a total of 14 studies from 13 articles were finally included with 9,463 patients and 11,416 controls [19]–[31]. For the R144C polymorphism (rs1799853), 13 studies were available, including a total of 9,154 cases and 10,900 controls. For the I359L polymorphism (rs1057910), 13 studies involved a total of 7,701 cases and 9,287 controls. These three polymorphisms were found to occur in frequencies consistent with HWE in the control populations of the vast majority of the published studies. Of the cases, 96% were Caucasian, 4% were other ethnic origins. The detailed characteristics of the studies included in this meta-analysis are shown in Table 1.

**Table 1:** Characteristics of the studies included in the meta-analysis.

Study	Year	Ethnicity	Case	Control	No. of case/control	Gender distributionin cases/controls(male %)	Control source	Genotyping method
Martínez [19]	2001	Spanish	Histology confirmed	Healthy	110/123	53.0/53.0	PB	RFLP
Chan [20]	2004	American	Histology confirmed	Healthy	339/350	0/0	PB	Taqman
Tranah [21]	2005	American	CRC patients	Healthy	416/825	0/0	PB	Taqman
Landi [22]	2005	Spanish	CRC patients	Cancer free	364/324	NA/NA	HB	Sequencing
McGreavey [23]	2005	British	ICD-9	Healthy	490/592	61.0/54.0	PB	Taqman
Samowitz [24]	2006	American	ICD-9	Healthy	2295/2903	57.1/54.6	PB	RFLP, TaqMan
Liao [25]	2007	Chinese	CRC patients	Healthy	284/483	54.5/53.6	PB	Sequencing
Küry [26]	2007	French	CRC patients	Healthy	1013/1118	62.0/54.0	PB	Taqman
Cotterchio [27]	2008	Canadian	ICD-9	Healthy	834/1249	NA/NA	PB	Taqman
Buyukdogan [28]	2009	Turkish	CRC patients	Healthy	77/78	52.0/53.0	PB	RT-PCR
Northwood [29]	2010	British	Colonoscopy confirmed	Healthy	308/296	71.3/58.8	PB	Taqman
Cleary [30]	2010	Canadian	ICD-9	Healthy	1165/1292	41.0/56.0	PB	Taqman
Sainz [31]	2011	German	CRC patients	Healthy	1768/1783	58.6/59.8	PB	KASPar assay

ICD: International Classification of Diseases, NA: not available, HB: hospital-based, PB: population-based.

### Association of R144C Polymorphism with CRC

**Figure 2:**
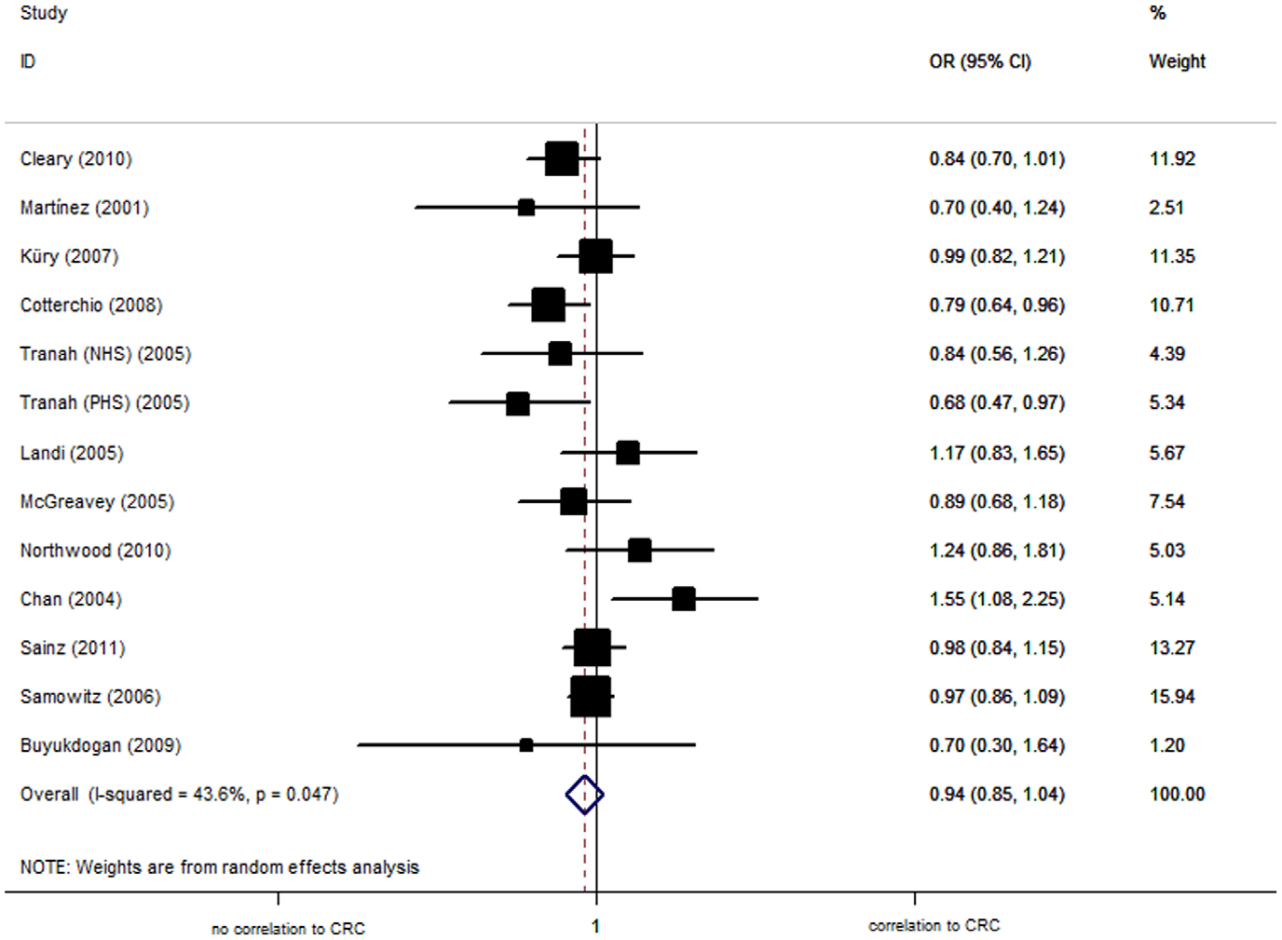
Forest plot from the meta-analysis of colorectal cancer and CYP2C9 R144C polymorphism using dominant genetic model.

Overall, there was no evidence of an association between the increased risk of CRC and the R144C variant in different genetic models when all eligible studies were pooled into the meta-analysis. Using random effect model, the per-allele overall OR of the C variant for CRC was 0.98 [95% CI: 0.89−1.06; *P*(Z) = 0.58; *P*(Q) = 0.04], with corresponding results under dominant and recessive genetic models of 0.94 [95% CI: 0.85−1.04; *P*(Z) = 0.21; *P*(Q) = 0.05, Figure 2] and 1.22 [95% CI: 1.00−1.47; *P*(Z) = 0.05; *P*(Q) = 0.46], respectively.

Analysis restricted to the 12 studies conducted in Caucasian population (total, 9077 cases and 10822 controls), yielded an per-allele OR for C variant of 0.98 [95% CI: 0.90−1.07; *P*(Z) = 0.16; *P*(Q) = 0.09]. Similar results were also found using dominant [OR = 0.94, 95% CI: 0.86−1.04; *P*(Z) = 0.25; *P*(Q) = 0.04] or recessive [OR = 1.24, 95% CI: 1.00−1.52; *P*(Z) = 0.05; *P*(Q) = 0.39] genetic model. The polymorphism was found to occur in frequencies consistent with HWE in the control populations for these studies.

The data on genotypes of the polymorphism among cases stratified by the sex were available in 5 studies. The per-allele OR was 1.06 [95% CI: 0.75−1.50; *P*(Z) = 0.73; *P*(Q) = 0.02] in woman compared to 0.91 [95% CI: 0.71−1.17; *P*(Z) = 0.45; *P*(Q) = 0.15] in men. Similarly, no statistically significant results were observed under dominant and recessive genetic models.

### Association of I359L Polymorphism with CRC

**Figure 3:**
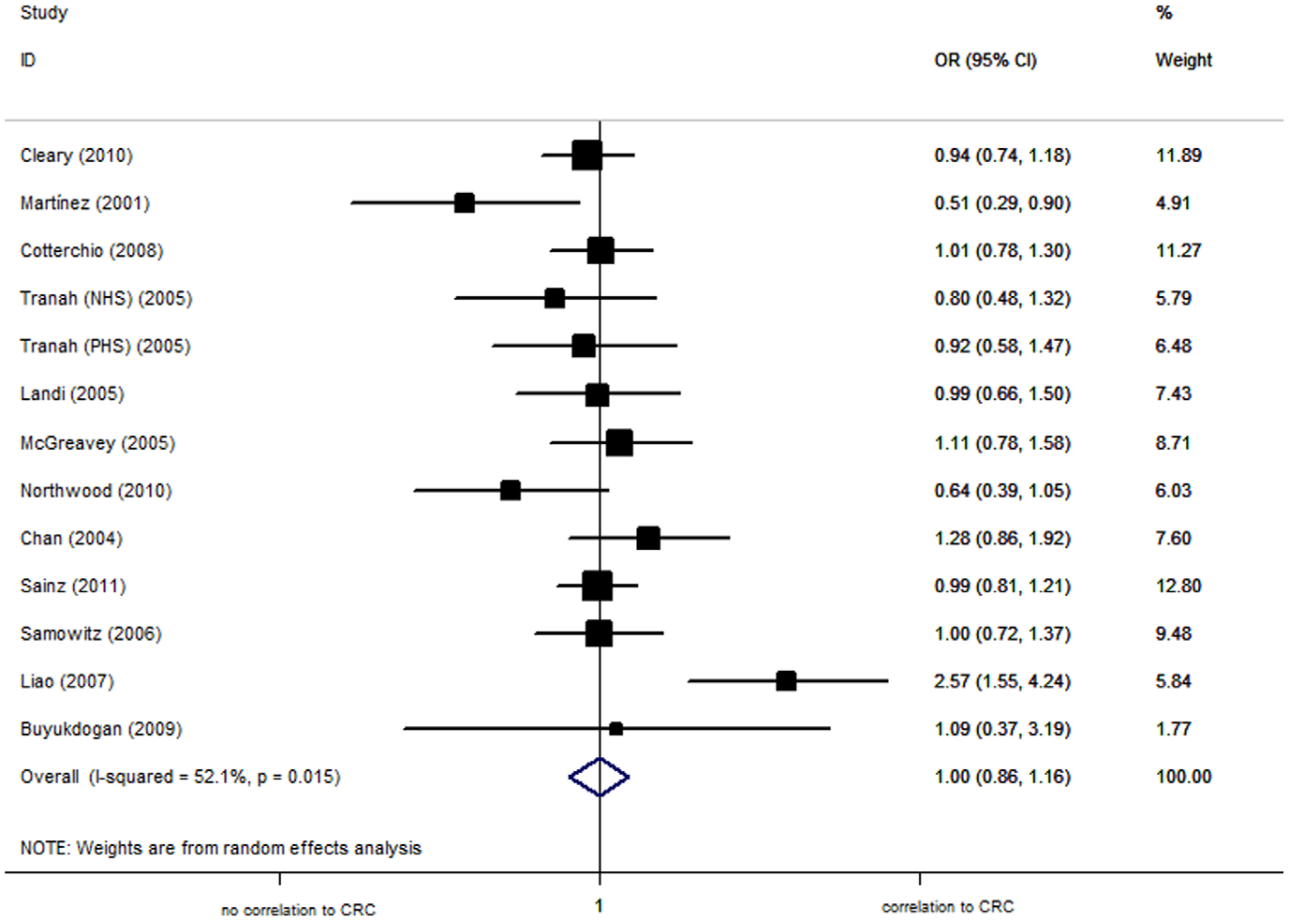
Forest plot from the meta-analysis of colorectal cancer and CYP2C9 I359L polymorphism using dominant genetic model.

Overall, the per-allele OR of the I359L polymorphism for CRC was 0.99 [95% CI: 0.87−1.14; *P*(Z) = 0.94; *P*(Q) = 0.03], with corresponding results under dominant and recessive genetic models of 1.00 [95% CI: 0.86−1.16; *P*(Z) = 0.97; *P*(Q) = 0.02, Figure 3] and 0.87 [95% CI: 0.41−1.88; *P*(Z) = 0.73; *P*(Q) = 0.21], respectively. One study founded to be deviated from HWE, negative results still maintained after excluding it [L allele: OR = 1.02, 95%CI = 0.90−1.16, *P*(Z) = 0.75, *P*(Q) = 0.07; dominant model: OR = 1.03, 95% CI = 0.89−1.18, *P*(Z) = 0.70, *P*(Q) = 0.05; recessive model: OR = 0.87, 95% CI = 0.40−1.88, *P*(Z) = 0.73, *P*(Q) = 0.21].

The meta-analysis included 11 studies (7,374 cases and 8,734 controls) in Caucasian population. The Q-test of heterogeneity was not significant in the contrasts of L versus I, dominant, and recessive genetic models. No statistically significant association was established for the CYP2C9 I359L polymorphism in Caucasian population (L allele: OR = 0.95, 95% CI = 0.87−1.05, *P* = 0.33; dominant model: OR = 0.96, 95% CI = 0.86−1.06, *P* = 0.40; recessive model: OR = 0.87, 95% CI = 0.40−1.88, *P* = 0.73).

When studies were stratified for sex (6 studies), no significant risks were found among men in all genetic models [L allele: OR = 1.22, 95% CI = 0.66−2.28, *P*(Z) = 0.54; dominant model: OR = 1.24, 95% CI = 0.64−2.41, *P*(Z) = 0.52; recessive model: OR = 0.69, 95% CI = 0.11−4.11, *P*(Z) = 0.68]. Similar results were also found in the woman [L allele: OR = 1.21, 95% CI = 0.94−1.57, *P*(Z) = 0.14; dominant model: OR = 1.22, 95% CI = 0.92−1.60, *P*(Z) = 0.16; recessive model: OR = 2.03, 95% CI = 0.51−8.15, *P*(Z) = 0.32].

### Sensitivity Analyses and Publication Bias

A single study involved in the meta-analysis was deleted each time to reflect the influence of the individual dataset to the pooled ORs, and the corresponding pooled ORs were not qualitatively altered (data not shown). Begg’s funnel plot and Egger’s test were performed to access the publication bias of the literatures. The shape of the funnel plots was symmetrical for the two polymorphisms (Figure S1 and S2). The statistical results still did not show publication bias in these studies for R144C (Egger’s test: *P* = 0.86) and I359L (Egger’s test: *P* = 0.97).

## Discussion

Large sample and unbiased epidemiological studies of predisposition genes polymorphisms could provide insight into the in vivo relationship between candidate genes and complex diseases. The involvement of CYP2C9 enzyme in the metabolism of xenobiotics could underlie the mechanism responsible for the association of CYP2C9 genotype and colorectal cancer. So far, many studies had focused on association between CYP2C9 polymorphism and CRC, but the results were still unclear. This is the first comprehensive meta-analysis examined the two functional polymorphism (R144C and I359L) of CYP2C9 and the relationship to susceptibility for CRC. Its strength was based on the accumulation of published data giving greater information to detect significant differences. In total, the meta-analysis involved 14 studies for CRC which provided 9,463 cases and 11,416 controls.

In this large-scale meta-analysis, the combined evidence suggested that CYP2C9 *2 and *3 polymorphism did not contribute to the development of CRC. However, CRC is a complex disease, and both environmental and genetic factors are involved in the development of CRC. There are some possible reasons for the inconsistent results in early reports. Firstly, ethnic differences may attribute to these different results, since the distributions of the CYP2C9 polymorphism were different between various ethnic populations. For instance, the frequencies of CYP2C9*2 polymorphism allele differs from 0.6% in Chinese population [32], 11% in British populations [29], to 18% in Caucasian-American [21]. On the other hand, study design or small sample size or some environmental factors may affect the results. Most of these studies did not consider most of the important environmental factors. It is possible that variation at this locus has modest effects on CRC, but environmental factors may predominate in the progress of CRC, and mask the effects of this variation. Specific environmental factors like lifestyle and cigarette smoking have already been well studied in recent decades [21], [26]. The unconsidered factors mixed together may cover the role of *CYP2C9* polymorphism. Thus, even if the variation has a causal effect on CRC, it may take a long time to be observed.

CYP2C9*3 encodes a protein with approximately 5–30% of the activity of the common reference allele [33], and it could therefore be hypothesized that CYP2C9*3 allele carriers have reduced carcinogen activating ability and thus reduced disease risk. However, the increased CRC risk associated with CYP2C9*2 genotype in earlier studies suggests that the enzyme may play a more important role in detoxification of carcinogens. The CYP2C9*3 association data should therefore be considered as a hypothesis-generating observation, which requires replication in larger independent cohorts. We also acknowledge that our study is not sufficiently powered to investigate the influence of low frequency alleles such as CYP2C9*3 (few CYP2C9*3/*3 homozygotes were present in our study) even by pooling all available data together. It is also important to note that CYP2C9, like many other P450 enzymes is inducible by a variety of structurally diverse chemicals, as part of the body’s adaptive response to environmental challenge [34]. It will therefore be of interest in future studies to investigate the extent of individuality in CYP2C9 protein expression and activity (and the expression of other inducible drug metabolizing enzymes), to assess the extent to which CYP2C9 phenotype influences CRC risk.

In interpreting the results, some limitations of this meta-analysis should be addressed. Firstly, our results were based on unadjusted estimates, while a more precise analysis should be conducted if all individual raw data were available, which would allow for the adjustment by other co-variants including age, drinking status, cigarette consumption, and other lifestyle. Secondly, the subgroup meta-analyses considering sex different between CYP2C9 polymorphisms and CRC risk, was performed on the basis of a fraction of all the possible data to be pooled, so selection bias may have occurred and our results may be overinflated. Nevertheless, the total number of subjects included in this part of the analysis comprises the largest sample size so far. Thirdly, only published studies were included in this meta-analysis. Therefore, publication bias may have occurred, even though the use of a statistical test did not show it.

To conclude, our meta-analysis did not support an association of the R144C and I359L polymorphism of CYP2C9 with CRC. The importance of these polymorphisms as a predictor of the risk of CRC is probably very small and the screening utility of this genetic variant in asymptomatic individuals may not be warranted. It is also known that the pathogenesis of CRC is complex and polygenetic in the vast majority of patients, with several genes, each with a small to moderate effect, acting individually, together or in association with important environmental determinants. Larger studies of different ethnic populations, especially strict selection of patients, well-matched controls, are needed to confirm our findings.

## Supporting Information

Figure S1 Begg’s funnel plot of CYP2C9 R144C polymorphism and colorectal cancer.
(TIF)

Figure S2 Begg’s funnel plot of CYP2C9 I359L polymorphism and colorectal cancer.
(TIF)

Checklist S1 (DOC)
