# Correction: Meta-Analysis of Cytochrome P-450 2C9 Polymorphism and Colorectal Cancer Risk

**DOI:** 10.1371/annotation/405a8737-be71-4ff1-8755-a3ccf11cb67f

**Published:** 2013-05-07

**Authors:** Shuo Liang, Jinsong Hu, Weijun Cao, Sanjun Cai

The name of the second author was spelled incorrectly. The correct name is: Jinsong Hu 

