# Supplementary figures and images for: Meta-Analysis of Cytochrome P-450 2C9 Polymorphism and Colorectal Cancer Risk
Source: PLoS One. 2012 Nov 7;7(11):e49134. doi: 10.1371/journal.pone.0049134 (PMC3492323; doi:10.1371/journal.pone.0049134)

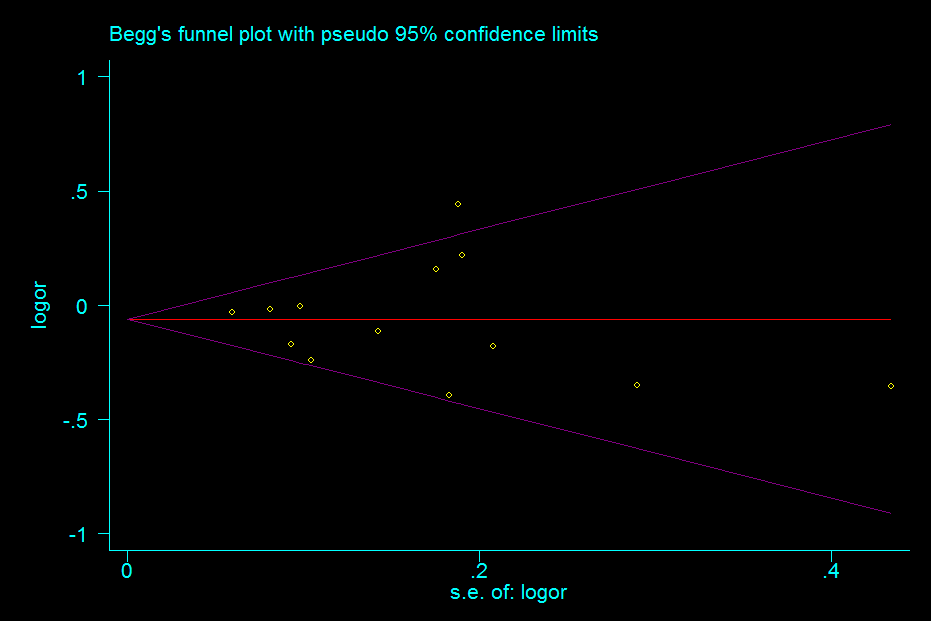

Supplement: Figure S1 — Begg’s funnel plot of CYP2C9 R144C polymorphism and colorectal cancer. (TIF) [file pone.0049134.s001.tif]

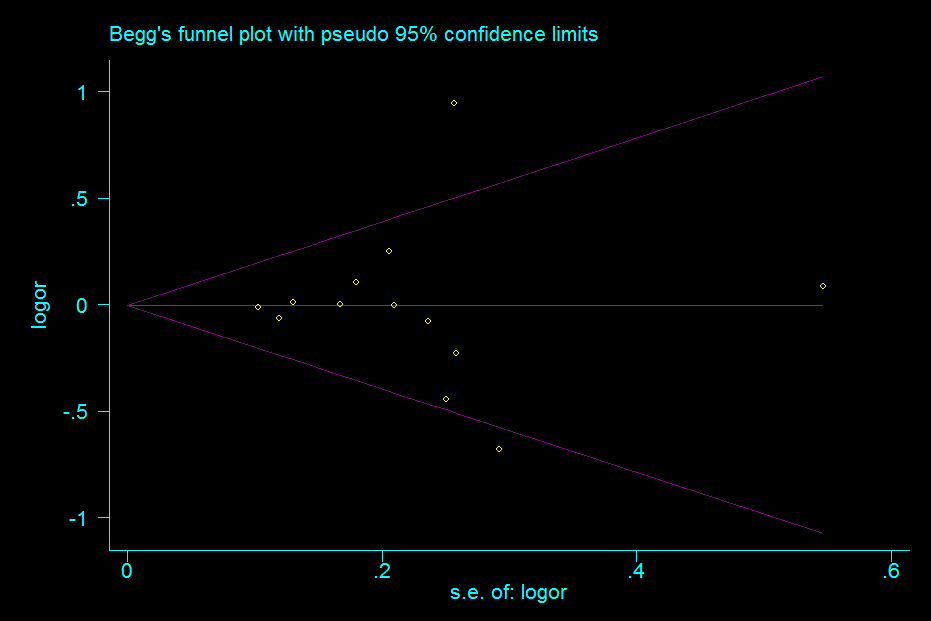

Supplement: Figure S2 — Begg’s funnel plot of CYP2C9 I359L polymorphism and colorectal cancer. (TIF) [file pone.0049134.s002.tif]
